# If Birds Have Sesamoid Bones, Do Blackbirds Have Sesamoid Bones? The Modification Effect With Known Compound Words

**DOI:** 10.3389/fpsyg.2019.01570

**Published:** 2019-07-09

**Authors:** Thomas L. Spalding, Christina L. Gagné, Kelly A. Nisbet, Jenna M. Chamberlain, Gary Libben

**Affiliations:** ^1^Department of Psychology, University of Alberta, Edmonton, AB, Canada; ^2^Department of Linguistics and Languages, McMaster University, Hamilton, ON, Canada; ^3^Department of Psychology, Brock University, St. Catharines, ON, Canada

**Keywords:** modification effect, compound words, modifier-noun phrases, property verification, concepts

## Abstract

Three experiments investigate how people infer properties of compound words from the unmodified head. Concepts license inference of properties true of the concept to instances or sub-types of that concept: Knowing that birds generally fly, one infers that a new type of bird flies. However, different names are also believed to reflect real underlying differences. Hence, a different name creates the expectation that a new bird differs from birds in general, and this might impact property inference. In these experiments, participants were told, Almost all (Some, Almost no) birds have sesamoid bones, and then asked, What percentage of blackbirds (birds) have sesamoid bones? The results indicate both inference and contrast effects. People infer properties as less common of the compound than the head when the property is true of the head, but they infer them as more common of the compound than the head when the property is not true of the head. In addition, inferences about properties true of the head are affected by the semantic similarity between the head and the compound, but properties not true of the head do not show any semantic similarity effect, but only a small, consistent effect of contrast. Finally, the presentation format (Open vs. Closed compounds) affects the pattern of effects only when the spacing suggests the existence of a permanent name.

## Introduction

Much research on compound words (words that consist of two or more free morphemes, e.g., *snowball* or *hogwash*) focuses on the processing involved in accessing the words (see, e.g., Libben, 1998; Libben and Jarema, 2006, for reviews) for use in specifically language-related tasks. There is much less work on how compound words are used more broadly in human cognition. In this paper, we investigate how compound words function in, and contribute to, human cognition more broadly. In particular, we are interested in what support compound words provide to conceptual (or categorical) inference. It is well accepted that a major function of concepts in human thinking is to provide the ability to infer properties from the concept to members of the category named or referred to by the concept (see e.g., Murphy, 2002; see also, Osherson et al., 1990). Thus, if one knows that birds have sesamoid bones, then one can make a reasonable inference that a particularnew bird has sesamoid bones, even though one has no other information that would specifically indicate this fact for this particular bird. Similarly, one might make a reasonable inference that blackbirds, in general, have sesamoid bones, if birds, in general, are believed to have sesamoid bones. Importantly, such inferences are probabilistic and defeasible, rather than strictly logical deductions (see Osherson et al., 1990), such that, for example, specific information about a particular item or sub-group may override the inference (e.g., knowledge that penguins cannot fly rules out the inference that penguins can fly, just because they are a kind of bird), as can the typicality of the sub-group with respect to the group (e.g., property inference is more likely from bird to robin than from bird to, say, turkey, even in the absence of knowledge about the specific property). However, the point of categorical inference is precisely that the category allows one to infer properties where there is no specific knowledge about the item or sub-group that can be brought to the question.

On the other hand, it is equally well accepted that people expect that different names for things reflect real underlying semantic differences. Indeed, the literature contains three proposed principles of human cognition (Synonomy Avoidance, Carstairs-McCarthy, 2010; Principle of Contrast, Clark, 1993; and Mutual Exclusivity Principle, Markman, 1989), each of which shares this core notion of expectation that different names reflect real underlying differences. While these principles are generally framed in terms of when a new name is, in some sense, justified, and how people make that decision, it is also the case that when a new name is presented as established, this leads to an expectation of some real differences from things that already have other names. Importantly, this expectation seems to be quite general, arising even when there is no specific knowledge of an existing difference. Taking these principles into account, then, one might expect that birds and blackbirds should be assumed to differ in significant ways, and this expectation might then affect the process of inferring properties from the head to the compound word. Thus, perhaps although birds generally do have sesamoid bones, blackbirds might not.

Clearly, then, we have two well-attested principles, which seem to work in opposite directions in terms of property inference: That categories license inference of properties to new sub-categories or category members, and that new labels, for example, of a sub-category, indicate property differences (i.e., a lack of licensed inference) for new sub-categories or category members. It is important to understand how these two principles operate together, as property inference is a major communicative function: Property inference (or lack thereof) creates expectations of novel objects, people, situations, and so on, based on what is known or said of an existing set, so that one is in a better position to deal with that novel object, person, or situation. This predictive function is also critical in numerous areas of applied work, for example in natural language processing, where understanding the expectations of a user is critical to the success of the system. As one example, research aimed at improving information retrieval engines (e.g., Baldwin et al., 2010; Wang et al., 2010) by identifying possible alternative terms that might be used to facilitate information access is one example where it is critical to understand what humans expect about the meaning of new terms. Similarly, understanding property inference can contribute to research on natural language question answering (for an overview, see Hirschman and Gaizauskas, 2001). For example, Wang et al. (2011) presented methods for identifying discourse structure for online forum data (see also Wang et al., 2010). The dialogue acts within these threads have various structures such as Question–Question, Answer–Answer, Question–Additional Information, and the meaning of terms in these dialog acts depends, in part, on the structure of those dialog acts (e.g., similar expressions can carry somewhat different meanings and referents, depending on whether they are embedded in an answer or in a request for additional information). Discovering the factors that influence the way in which the expression is used to refer to a referent and the properties of that referent can aid the development of NLP procedures used to automate the identification of dialogue acts. In addition, systems that attempt to automatically extract sentiment must be built keeping in mind the ways in which people infer properties of objects as existing labels are used and new labels are introduced (e.g., Maynard and Funk, 2012; Dragos et al., 2018). In sum, understanding how property inference operates in light of these two contrasting principles has important scientific, and also applied, consequences.

We begin by reviewing what is known about how people extend properties of concepts to novel combined concepts, and then turn to the question of whether people extend properties to known compound words in the same way. The question of when and which properties of a combined concept become available during conceptual combination has been one of the core questions within the conceptual combination literature. Research on this topic initially examined whether properties of the constituent concepts are available prior to properties of the whole concept. Early research by Springer and Murphy (1992) found that people were faster to verify properties that were true of the phrase (e.g., peeled apples are white) than properties that were true of the head concept, prior to modification (e.g., peeled apples are round). Gagné and Murphy (1996) found that discourse context did not alter this pattern.

More recently, the question of property inclusion has been examined in the context of examining whether the availability of properties differs for the head noun concept (e.g., ducks have webbed feet) relative to a modified concept (e.g., baby ducks have webbed feet). When using novel combinations, this work reveals a robust set of effects called the modification and inverse modification effects (see Spalding and Gagné, 2015, for a demonstration of both modification and inverse modification effects, but for modification effects using other property verification tasks see also Connolly et al., 2007; Jönsson and Hampton, 2008, 2012; Gagné and Spalding, 2011, 2014b; Hampton et al., 2011). In particular, properties generally true of the unmodified head noun become less true of the modified head (modification effect), while properties generally false of the unmodified head become less false of the modified head (inverse modification effect). Thus, for example, purple candles are judged less likely to be made of wax than candles, but purple candles are also judged more likely to have teeth than candles. The modification effect is robust over a range of specific verification tasks, including ratings of likelihood of the truth/plausibility of a property for a category (e.g., Connolly et al., 2007), true/false decisions about the property’s relation to the concept and the response times to make those decisions (e.g., Gagné and Spalding, 2011), and estimates of the percentages of category members for which the property is true (e.g., Spalding and Gagné, 2015). Also, the modification effect is very robust over a wide range of property typicality, including properties that seem to be nearly definitional of the head, such as being animate for lamb (see e.g., Jönsson and Hampton, 2008, 2012; Hampton et al., 2011). This robustness over various kinds of properties is unexpected by those theories where prototypicality of features should be a determining aspect of property verification, such as prototype theories of conceptual combination (e.g., Hampton, 1991), but also theories of the semantics of compound words which differentiate between “levels” of properties (e.g., the skeleton vs. body distinction in Lieber, 2004).

Although the modification effect was initially used to test hypotheses about whether or not properties of a combined concept are directly inherited from the constituent concepts (e.g., Connolly et al., 2007), there are some findings to suggest that this effect might not actually be driven by the process of conceptual combination (i.e., constructing a new concept based on the conceptual “contents” of the modifier and head) *per se*, but rather by reasoning about the combined concepts. Gagné and Spalding (2011, 2014b, 2015), Spalding and Gagné (2015), Gagné et al. (2017) present evidence that the modification effects primarily arise due to meta-knowledge of modification, and particularly to reasoning about the expected relation of the meaning of the combined concept and the head. In particular, they argue that the effects are largely driven by the expected level of contrast (i.e., matching or mismatching features) between the combined concept and the head, rather than by conceptual knowledge of the individual constituent concepts (as would be expected by e.g., Hampton, 1987, 1991; but also by many approaches to the semantics of known compound words such as Lieber, 2004, 2009; see Gagné and Spalding, 2015, for a discussion). For example, modification effects arise even when the modifier is a non-word, and thus cannot contribute any semantic or conceptual information about what properties are appropriate for the combined concept (e.g., Spalding and Gagné, 2015).

In short, the literature on the modification effect strongly suggests that (a) the inferential function of concepts does indeed extend to modified versions of those concepts, rather than only to individual members of the category picked out by the concept, but that (b) the modification and inverse modification effects are driven by people’s expectations about the nature, purpose, and use of modification. Thus, it seems likely that the general principle that a different name implies other, underlying differences (Synonomy Avoidance, Carstairs-McCarthy, 2010; Principle of Contrast, Clark, 1993; and Mutual Exclusivity Principle, Markman, 1989) should lead to modification and inverse modification effects with known compounds. However, in the existing literature, the “different names” created by modification are not well-established, but rather are novel. This novelty could have two different kinds of influence on the modification effect. It could be that novel names are simply seen as less established or less permanent names, and therefore they might lead to smaller modification effects (i.e., more likelihood of property inference), or it could be that the novelty makes the contrast more immediately salient, and therefore leads to larger modification effects (i.e., less likelihood of property inference). Furthermore, because of the novelty of the modified concepts, the existing literature is unable to investigate the way in which these principles interact with the semantic knowledge that is inherent in a category of things with a well-established name, or, indeed, with the simple fact of the well-established name. Nevertheless, there is good reason to believe that much of the processing and semantics of compound words is similar to that of novel conceptual combinations (see, e.g., Gagné and Spalding, 2014a). Thus, we expect to see modification effects (and inverse modification effects) with known compounds.

In the current experiments, we investigate the seeming conflict between the basic cognitive principles that we infer properties based on category membership and that the use of different names for things implies real underlying differences (Synonomy Avoidance, Carstairs-McCarthy, 2010; Principle of Contrast, Clark, 1993; and Mutual Exclusivity Principle, Markman, 1989), in three experiments. In particular, we investigate the extent to which people are willing to infer properties from head nouns to compounds under various conditions that should affect the extent to which people believe the compounds to be well-established as different names, and thus should bring the principle that different names imply different properties more into conflict with the principle that properties can be inferred from categories to more specific sub-sets. Experiment 1 investigates whether the modification and inverse modification effects occur for known, transparent compound words and further investigates whether semantic knowledge of the compounds and their relation to the head nouns plays a role. Experiment 2 replicates Experiment 1, and in addition investigates the effect of spacing, under the assumption that the lack of spacing would indicate the compounds as more established, permanent, unique names for existing sub-categories. Experiment 3 is similar to Experiment 2, except that we replaced the modifier of the compound with a non-word, in order to find out if the structural cue (spacing) would affect property inference when the “compound” is not a known category.

## Experiment 1

This experiment investigates how people infer properties of transparent compound words based on their knowledge of those properties’ relationship with the head noun, using the method from Spalding and Gagné (2015). This method asks participants to estimate the percentage of members of a category (e.g., buds) or subcategory (e.g., rosebuds) that have a particular property.

To directly manipulate the truth value of the property, we used blank predicates (i.e., properties that use relatively unfamiliar terms but are relevant for the concept in question, e.g., biological predicates are used for animal categories, see Osherson et al., 1990). The likelihood of the property for the unmodified noun was manipulated by telling participants that Almost All, Some, or Almost No members of the head noun concept had it. Critically, because we used the same blank properties in the Almost all, Some, and Almost No conditions, not only is the semantic content of those properties relatively unfamiliar and/or unrelated to the compounds, but to the extent it is familiar, the semantic content is controlled across the likelihood manipulation.

Based on previous work showing that processing compound words appears to involve many of the same processes as the processing of novel combined concepts (e.g., Gagné and Spalding, 2009), we predict that in making a decision about whether a property of the head is true of a compound word, people will show a pattern similar to that previously demonstrated for novel modifier-noun pairings: Modification effect for properties true of the head and inverse modification effect for properties false of the head.

### Methods

This and the following experiments were reviewed for ethical content and approved by the Research Ethics Committee at the University of Alberta, and all participants provided written consent.

#### Participants

Sixty-two participants took part in the study. Participants in this and all following experiments were undergraduate students enrolled in a very large first year psychology class and obtained partial class credit for participating. In this participant population, approximately 95% are between 18 and 24 years of age and approximately 70% are female. All participants in this and the following experiments self-identify as native English speakers. In this, and the following experiments, target sample size is determined by expected effect size and complexity of design. The number of actual participants is determined by the target sample size and availability of participants in the Departmental participant pool. In this experiment, we set a target of 10 participants per condition. Two extra participants were included (in our pool, if extra participants attend a session, they must be run).

#### Materials and Design

Experiment 1 used 96 transparent compounds (e.g., *snakeskin*) selected from a previously categorized set of items (Ji et al., 2011). Statements to be predicated of the heads of the compounds were then selected to match each compound word. The truth of these statements, relative to the compound words or the head nouns of the compound words, were expected to be unknown by the participants. The unknown predicates were taken from previous experiments on the modification effect (Spalding and Gagné, 2015) and from Wikipedia searches for uncommon words related to the head noun. The compounds and the predicates are presented in the Appendix. The design is a 2 (Modification: modified vs. unmodified) by 3 (Likelihood: almost all, some, almost no) crossed factorial design.

#### Procedure

On each trial, participants were first shown a statement regarding how often an unknown property is true of an unmodified noun using one of three quantifiers: Almost all, Some, or Almost no. For example, participants might see “Almost all birds require graminoids in their diet.” The participants were instructed to treat this statement as true. Participants were then asked a follow up question about how many members of the unmodified noun or the modified noun category have that same property. They were asked to respond on a scale of 1–100. For example, they would be asked either “What percentage of birds require graminoids in their diet?” or “What percentage of blackbirds require graminoids in their diet?”. The lists were counterbalanced so that each participant saw either the compound word or the head noun in the question.

### Results and Discussion

#### Data Analysis

The descriptive statistics are shown in Table 1. We analyzed the data using linear-mixed effects (LME) regression models in which subject and item were entered as random effects, and Modification (modified vs. unmodified) and Likelihood (Almost all, Some, Almost no) were entered as fixed effects, using the *mixed* and *contrast* commands in Stata (StataCorp, 2017). The *mixed* function outputs coefficients (i.e., estimates) for simple effects at the first level of the other categorical variables and at the mean of the other continuous variables in the model (see Table 2). For testing our hypotheses, these coefficients are not directly interpretable because they represent the simple effect of a variable at the first level of other variables. The relevant statistical tests for addressing our research questions concern interactions and simple effects, which are reported in the following text. The *contrast* function in Stata was used to conduct these analyses. We report the tests conducted on these fixed effects. Tests of simple effects (the effect of a factor at one level of another factor) were conducted to follow up on statistically significant interactions, because in the case of significant interactions, the main effects are not informative. Some statistics packages report LME main effects as *F*-tests and the simple effects as *t*-tests. However, because the degrees of freedom are indeterminate for such tests in linear mixed effect models, Stata uses chi-square and *Z*-scores, respectively.

**Table 1:** Mean (SE) judged percentage of category members having the test property by level of Likelihood from Experiment 1.

	Percent of category members (SE)		
Condition	Almost All	Some	Almost No
Unmodified	91.1 (1.5)	37.6 (2.0)	7.2 (1.4)
Modified	66.7 (4.7)	28.2 (3.0)	9.8 (2.3)


**Table 2:** Experiment 1 Mixed Model Coefficients.

Variable	Coefficient	Standard Error	*z*	*P* > |z|
Likelihood: Almost no	–56.8	0.916	–62.0	0.000
Likelihood: Some	–38.6	0.918	–42.1	0.000
Modification	24.4	0.909	26.9	0.000
Mod × Likelihood: Almost no	–27.0	1.29	–20.8	0.000
Mod × Likelihood: Some	–15.0	1.30	–11.6	0.000
Constant	66.7	1.03	65.0	0.000
Subjects	31.8	6.44		
Items	13.0	2.85		


#### Results

The analysis indicated a significant interaction between Modification and Likelihood, *X^2^*(2) = 435.0, *p* < 0.001. Analysis of the simple effects indicated that the Almost All and Some conditions both led to a significant modification effect, *z* = 26.9, *p* < 0.001 and *z* = 10.1, *p* < 0.001, respectively, while the Almost No condition led to a significant inverse modification effect, *z* = -2.8, *p* < 0.01. That is, the property was judged less likely for the compound, if the property was presented as true of almost all or some of the head category members, but more likely for the compound, if the property was presented as true of almost no members of the head category. Clearly, known compound words give rise to a very robust modification and inverse modification effect, as predicted.

These results show that modification and inverse modification effects that have been reported for novel phrases extend to lexicalized compounds. However, there are several more specific points that should be noted. First, the modification effects are much larger than those found in previous studies with novel phrases. For example, in the current experiment, modification in the Almost All condition reduced the likelihood of the property by some 24 percentage points, while Spalding and Gagné (2015) found a reduction of only 2 percentage points, using exactly the same experimental paradigm and participant population. This is consistent with the notion that the degree to which the compound word is seen as a unique, permanent, established name is likely to make people believe more strongly that there are real, underlying differences between the things named by the head and by the compound. Second, there is a strong asymmetry between the true and false features (i.e., between the size of the modification and inverse modification effects), suggesting that the existence of a known compound affects the features presented as generally false of the head noun much less than those presented as generally true of the head.

**FIGURE 1:**
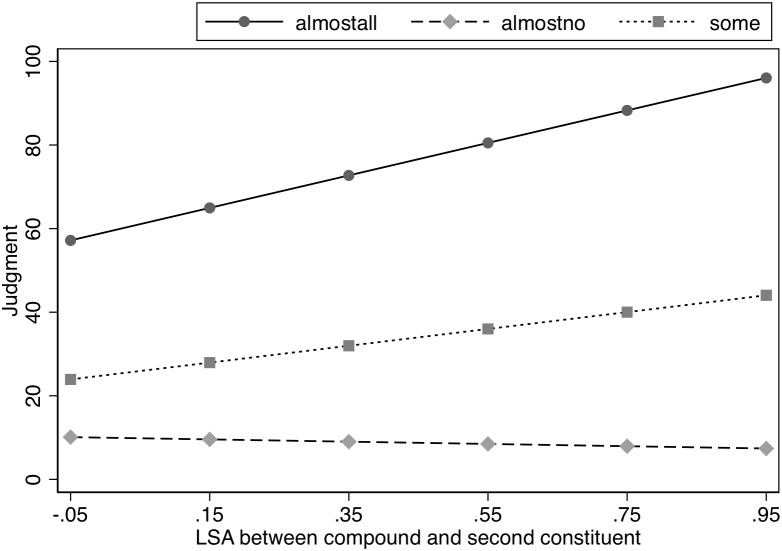
Effect of semantic similarity (LSA) on judged likelihood of property in Experiment 1.

To investigate the pattern of asymmetry between the true and false features further, and to investigate the role of the semantics of the existing compound word, we performed a *post hoc* analysis, in which we entered the semantic similarity between the known compound and the unmodified head, as measured by Latent Semantic Analysis (LSA; Landauer and Dumais, 1997), into an LME regression with the Likelihood factor (Almost All, Some, Almost No), using only the percentage estimates for the Modified condition (see Table 3). Given that the compound was never seen in the unmodified condition, it would not be meaningful to include these items when examining the impact of the similarity between the compound and head. That is, the unmodified condition is when the head is presented for the property judgment, thus those data points reflect the judgment made when the participant is not presented with the compound word, and hence the semantic similarity between the compound and head is not meaningful in these cases (e.g., if the participant judges the likelihood that birds require graminoids in their diet, they will not have seen blackbird in the experiment at all, and hence the relationship between blackbird and bird is completely irrelevant). We observed a significant interaction between the LSA measure (e.g., the association between the word *birds* and the word *blackbirds*) and Likelihood, χ^2^(2) = 38.2, *p* < 0.001. Further analysis indicated a significant slope for LSA in the Almost All and Some conditions, *z* = 5.8, *p* < 0.001 and *z* = 3.0, *p* < 0.005, respectively. However, the semantic similarity between the compound and the unmodified head did not affect the estimates in the Almost No condition, *z* < 1. See Figure 1. In short, the modification effects were significantly smaller for compounds that were more semantically similar to their heads in the Almost All and Some conditions, but semantic similarity between the compound and the unmodified head had no effect in the Almost No condition.

**Table 3:** Experiment 1 Mixed Model Coefficients with LSA: Modified condition only.

Variable	Coefficient	Standard Error	*z*	*P* > |z|
Likelihood: Almost no	–49.2	6.68	–24.8	0.000
Likelihood: Some	–34.2	1.98	–17.4	0.000
LSA	38.9	6.68	5.8	0.000
LSA × Likelihood: Almost no	–41.6	6.74	–6.2	0.000
LSA × Likelihood: Some	–18.8	6.94	–2.7	0.007
Constant	59.1	2.16	27.4	0.000
Subjects	2.1	0.12		
Items	2.0	0.12		


**FIGURE 2:**
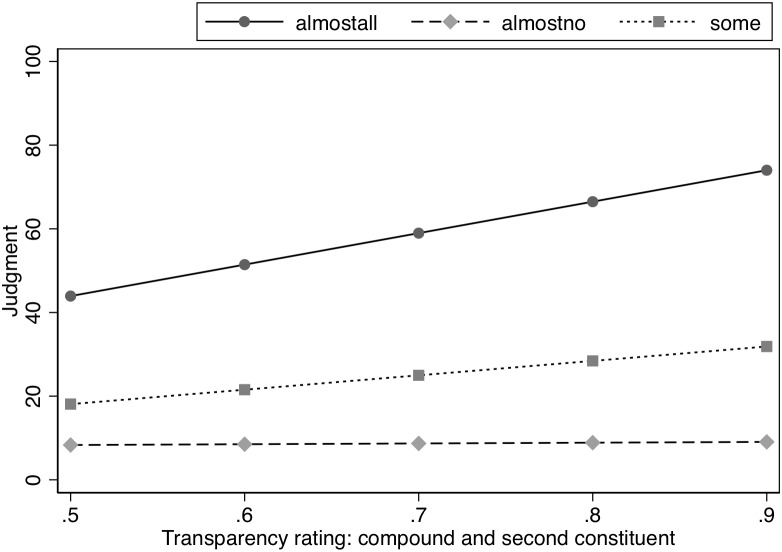
Effect of semantic similarity (human ratings) on judged likelihood of property in Experiment 1.

To further examine whether property inference is influenced by degree of semantic transparency, we also used semantic transparency ratings by human participants from a database containing semantic transparency ratings for over 8000 compounds (Gagné et al., in press). The judgment that is relevant for the current experiment is the rating between the head noun and the compound. Participants were asked to judge on a scale from 0 to 100% how much the head noun retained its meaning in the compound (e.g., *How much is the meaning of birds* *retained in the meaning of* *blackbirds*). This information was available for 71 items. The mean rating was 79% (*SD* = 13) and ranged from 52 to 96%. This judgment was entered into a model that also included Likelihood and was restricted to only the modified concept condition (see Table 4). There was an interaction between transparency rating and Likelihood, χ^2^(2) = 49.99, *p* < 0.0001. Analysis of this interaction indicated a significant slope for the transparency rating in the Almost All and Some conditions, *z* = 7.77, *p* < 0.0001 and *z* = 3.32, *p* < 0.002, respectively, with the slope for transparency judgments being steeper in the Almost All condition than in the Some condition, χ^2^(1) = 13.83, *p* < 0.001. As transparency increased, ratings for the properties increased for the modified items, meaning that more transparency would correspond to smaller modification effects. However, transparency judgments did not affect the estimates in the Almost No condition, *z* = 0.19, *p* = 0.852. See Figure 2.

**Table 4:** Experiment 1 Mixed Model Coefficients with Semantic Transparency judgments: Modified condition only.

Variable	Coefficient	Standard Error	*z*	*P* > |z|
Likelihood: Almost no	1.1	8.3	0.1	0.900
Likelihood: Some	–5.4	8.7	–0.6	0.534
Semantic Transparency	75.2	9.7	7.8	0.000
ST × Likelihood: Almost no	–73.4	10.4	–7.1	0.000
ST × Likelihood: Some	–40.8	11.0	–43.7	0.000
Constant	6.3	7.8	0.81	0.416
Subjects	2.1	0.11		
Items	1.9	0.13		


#### Discussion

Although the specific properties being tested are unrelated to participants’ existing knowledge of the known compound, properties presented as true or somewhat true of the head are sensitive to the overall known semantic difference between the compound and the head. Thus, Experiment 1 shows clearly that, in addition to an expectation of some contrast due to the existence of the compound as a name that contrasts in some way with the head, the semantics of the specific compound word also contribute to the modification effects with known compound words when Almost All or Some members of the head noun have that property.

However, when the properties are presented as false of the head (i.e., Almost No members of the head noun have the property) the participants are insensitive to the degree of semantic difference between the compound and the head, indicating that the inverse modification effect with such features might provide us with a kind of baseline measure of the pure effect of expectation of contrast driven purely by the fact of the different name (i.e., the fact that there is an existing compound that contrasts with the head). We propose that this difference stems from meta-knowledge about the relationship between properties and concepts. True properties are assumed to be related to the semantics of the concept and false properties are not (see e.g., Murphy and Medin, 1985, for discussion of what features are relevant to a given concept). That is, our concepts, in general, tend to be organized around things that are true of them (e.g., we tend to think of birds, for example, more in terms of the fact that they usually fly, have wings, and have feathers, rather than in terms of the fact that they do not usually explode or earn PhD’s).

Thus, true properties are influenced by the actual (pre-existing) semantic similarity between the compound and head because this similarity is used as one source of information about the expected level of contrast when making property judgments in the Almost All and Some conditions. On the other hand, in the Almost No condition, semantic similarity is not seen as being relevant due to the meta-knowledge that false properties are not generally associated with the concepts. We return to this point in the section “General Discussion.”

## Experiment 2

Experiment 1 showed very robust modification and inverse modification effects, with a strong asymmetry between the size of the two effects. In this experiment, we attempt to replicate these effects. Further, we examine whether property inferences for known compounds are affected by presenting the compounds with an open structure (e.g., *black bird*) or a closed structure (e.g., *blackbird*). This comparison will allow us to determine whether a compound (i.e., closed) structure encourages people to view the modified concept as being more distinct from the head noun concept than does a phrase-like structure, when the items are known compounds. On one hand, given that (in English), a closed compound structure is associated with more established compound words (see e.g., Kuperman and Bertram, 2013), if these effects are primarily driven by the notion that anything with a separate, existing, established compound name should have semantic differences from the head noun, one might expect that the open presentation would decrease the expectation of those semantic differences (and thus, an open presentation should attenuate the modification and inverse modification effects). On the other hand, simply presenting these well-known compound words with a space is, perhaps, unlikely to overcome the participants’ knowledge that these are, in fact, established compound words. Thus, spacing might not be influential, because the participants might be unlikely to believe that the inserted space indicates a novel phrase.

### Methods

#### Participants

Hundred and sixty three participants took part in the experiment. Each of the 12 lists was seen by a minimum of 13 and a maximum of 15 participants. We set a target of 15 participants per condition, but were not able to test the full number. We initially set a larger target per condition than in Experiment 1 because the design is more complicated. However, the effect sizes are quite large, and the sample size we obtained is more than sufficient.

#### Materials and Design

As in the previous experiment, we manipulated whether the concept was modified or unmodified, and the likelihood of the property (e.g., Some, Almost All, and Almost No). Modification and Likelihood were within-subject variables and were counterbalanced into 6 lists as in Experiment 1. The materials were identical to those used in Experiment 1. We also manipulated whether the modified concept was presented as a closed compound (e.g., *blackbird*) as in Experiment 1, or as an open compound (e.g., *black bird).* Spacing was a between-subjects factor to avoid drawing attention to this factor of interest and, thus, there were 12 lists (six with open items and six with closed items). Each participant saw one list. The design was a 2 (Modification) by 3 (Likelihood) by 2 (Spacing) crossed factorial design.

#### Procedure

The procedure was identical to Experiment 1.

### Results and Discussion

#### Data Analysis

The descriptive statistics are shown in Table 5. We analyzed the data using LME regression models in which subject and item were entered as crossed random effects, and Modification (modified vs. unmodified), Likelihood (Almost All, Some, Almost No) and Spacing (open vs. closed) were entered as fixed effects, using the mixed and contrast commands in Stata (StataCorp, 2017). The *mixed* function outputs coefficients (i.e., estimates) for simple effects at the first level of the other categorical variables and at the mean of the other continuous variables in the model (see Table 6). For testing our hypotheses, these coefficients are not directly interpretable because they represent the simple effect of a variable at the first level of other variables. The relevant statistical tests for addressing our research questions concern interactions and simple effects, which are reported in the following text. The *contrast* function in Stata was used to conduct these analyses. We report the tests conducted on these fixed effects. Tests of simple effects (the effect of a factor at one level of another factor) were conducted to follow up on statistically significant interactions, because in the case of significant interactions, the main effects are not informative.

**Table 5:** Mean (SE) judged percentage of category members having the test property by level of Likelihood from Experiment 2.

	Percent of category members (*SE*)			
Spacing	Condition	Almost All	Some	Almost No
Closed	Unmodified	91.1 (0.24)	35.5 (0.41)	7.3 (0.25)
	Modified	64.9 (1.04)	27.3 (0.61)	11.0 (0.54)
Open	Unmodified	88.6 (0.37)	40.3 (0.47	8.8 (0.37)
	Modified	65.7 (0.97)	32.7 (0.68)	13.5 (0.57)


**Table 6:** Experiment 2 Mixed Model Coefficients.

Variable	Coefficient	Standard Error	*z*	*P* > |z|
Spacing	0.8	1.2	0.7	0.481
Likelihood: Almost no	–53.9	0.81	–66.6	0.000
Likelihood: Some	–37.6	0.81	–46.5	0.000
Spacing × Likelihood Almost no	–1.7	1.13	1.5	0.130
Spacing × Likelihood Some	4.6	1.13	4.1	0.000
Modification	26.2	0.81	32.4	0.000
Spacing × Modification	–3.3	1.13	–2.9	0.003
Mod × Likelihood: Almost no	–29.9	1.14	–26.1	0.000
Mod × Likelihood: Some	–18.0	1.14	–15.8	0.000
Spacing × Mod × Likelihood: Almost no	2.2	1.59	1.4	0.162
Spacing × Mod × Likelihood: Some	2.7	1.59	1.7	0.097
Constant	64.9	0.94	69.1	0.000
Subjects	1.7	0.06		
Items	1.3	0.09		


#### Results

As in Experiment 1, we found modification and inverse modification effects. There was a significant interaction between Modification and Likelihood, χ^2^(2) = 1314.37, *p* < 0.00001. The tests of the simple effects revealed that the Almost All and Some conditions both led to a significant modification effect, *z* = 43.48, *p* < 0.0001 and *z* = 13.87, *p* < 0.0001, respectively, while the Almost No condition led to a significant inverse modification effect, *z* = -7.53, *p* < 0.0001. These effects were not affected by Spacing (e.g., *blackbird* vs. *black bird*); the three-way interaction between Spacing, Modification, and Likelihood was not significant, χ^2^(2) = 3.19, *p* = 0.20. Thus, in the case of known compounds, both the open and closed structure produced the same size of modification (or inverse modification) effects.

Although Spacing did not influence the two-way interaction between Modification and Likelihood (as indicated by the lack of three-way interaction), it did interact with Likelihood, χ*^2^*(2) = 55.19, *p* < 0.0001. In the Some condition, open items received higher ratings (*M* = 36.5, *SE* = 0.83) than did the closed items (*M* = 31.4, *SE* = 0.85), *z* = 4.84, *p* < 0.0001. However, spacing did not have an effect in the Almost All condition, *z* < 1. Influence of spacing in the Almost No condition was marginally significant, z = 1.9, *p* = 0.06, with ratings for open items being slightly higher (*M* = 11.1, *SE* = 0.83) than for closed items (*M* = 9.11, *SE* = 0.85). In sum, participants who received the open items gave higher ratings for the Some condition, whether the concept was modified or not, and, thus, this increase did not influence the modification effect itself.

Spacing also interacted with Modification, χ^2^(1) = 6.62, *p* < 0.01. As expected given that the compound was never seen in the unmodified condition, spacing did not affect the unmodified items, *z* = 1.26, *p* = 0.21. For the modified items, open items received higher ratings (*M* = 45.7, *SE* = 0.80) than did closed items (*M* = 34.4, *SE* = 0.81), *z* = 2.93, *p* = 0.003. To illustrate, participants were more willing to attribute an unknown property when the combined concept was expressed as an open form (*black bird*) than when it was a closed form (e.g., *blackbird*).

**FIGURE 3:**
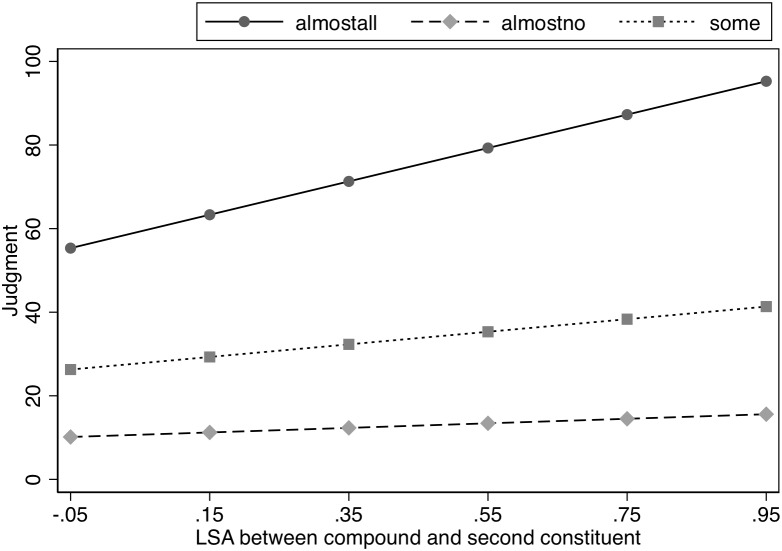
Effect of semantic similarity (LSA) on judged likelihood of property in Experiment 2.

To further explore the influence of semantic transparency, as in Experiment 1, we included a *post hoc* examination of whether the similarity between the head and the compound influenced the ratings by including the LSA measure for the head and the compound in a model that also included Spacing and Likelihood (see Table 7). As in Experiment 1, the analysis was restricted to only the modified concept condition because the compound was never seen in the unmodified condition and, thus, it would not be meaningful to include these items when examining the impact of the similarity between the compound and head. Spacing did not influence the nature of the interaction between LSA and Likelihood as indicated by the lack of interaction between these three variables, χ^2^(2) = 1.24, *p* = 0.54. As in Experiment 1, there was a significant interaction between the LSA measure and Likelihood, χ*^2^*(2) = 73.64, *p* < 0.0001. Further analysis indicated a significant slope for LSA in the Almost All and Some conditions, *z* = 7.71, *p* < 0.0001 and *z* = 2.91, *p* < 0.004, respectively, with the slope for transparency ratings being steeper in the Almost All condition than in the Some condition, χ*^2^*(1) = 35.07, *p* < 0.0001. However, the semantic association (as indicated by LSA) between the compound and the unmodified head did not affect the estimates in the Almost No condition, *z* = 1.05, *p* = 0.29. In sum, exactly as in Experiment 1, the modification effects were significantly smaller for compounds that were more semantically similar to their heads in the Almost all and Some conditions, but semantic similarity between the compound and the unmodified head had no effect in the Almost No condition. See Figure 3.

**Table 7:** Experiment 2 Mixed Model Coefficients with LSA: Modified condition only.

Variable	Coefficient	Standard Error	*z*	*P* > |z|
Spacing	1.7	2.2	0.8	0.418
Likelihood: Almost no	–47.0	1.7	–27.5	0.000
Likelihood: Some	–32.4	1.7	–18.9	0.000
Spacing × Likelihood Almost no	0.2	2.4	0.8	0.943
Spacing × Likelihood Some	4.0	2.4	1.7	0.094
LSA	42.8	6.0	7.1	0.000
Spacing × LSA	–5.5	5.9	–0.9	0.353
LSA × Likelihood: Almost no	–39.2	6.0	–6.6	0.000
LSA × Likelihood: Some	–28.0	6.0	–4.6	0.000
Spacing × LSA × Likelihood: Almost no	9.1	8.3	1.1	0.272
Spacing × LSA × Likelihood: Some	5.9	8.4	0.71	0.480
Constant	56.4	2.0	28.7	0.000
Subjects	2.1	0.07		
Items	1.9	0.10		


**FIGURE 4:**
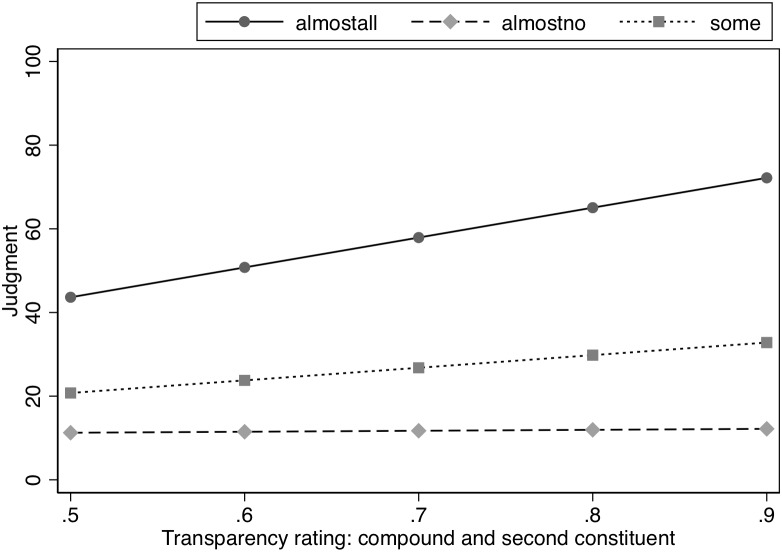
Effect of semantic similarity (human ratings) on judged likelihood of property in Experiment 2.

As in Experiment 1, to further examine whether property inference is influenced by degree of semantic transparency, we also used semantic transparency ratings by human participants from a database containing semantic transparency ratings for over 8000 compounds (Gagné et al., in press). The judgment of how much meaning of the head was retained in the compound was entered into a model that also included Spacing and Likelihood and was restricted to only the modified concept condition (see Table 8). Spacing did not influence the nature of the interaction between Transparency judgments and Likelihood as indicated by the lack of interaction between these three variables, χ^2^(2) = 0.50, *p* = 0.78. However, there was an interaction between transparency rating and Likelihood, χ^2^(2) = 117.67, *p* < 0.0001. Analysis of this interaction indicated a significant slope for the transparency rating in the Almost All and Some conditions, *z* = 9.54, *p* < 0.0001 and *z* = 4.02, *p* < 0.001, respectively, with the slope for transparency judgments being steeper in the Almost All condition than in the Some condition, χ*^2^*(1) = 40.95, *p* < 0.0001. As transparency increased, ratings for the properties increased for the modified items, meaning that more transparency would correspond to smaller modification effects. However, transparency judgments did not affect the estimates in the Almost No condition, *z* = 0.31, *p* = 0.76. See Figure 4.

**Table 8:** Experiment 2 Mixed Model Coefficients with Semantic Transparency judgments: Modified condition only.

Variable	Coefficient	Standard Error	*z*	*P* > |z|
Spacing	4.8	7.3	0.7	0.511
Likelihood: Almost no	3.3	7.3	0.4	0.657
Likelihood: Some	–5.9	7.4	–0.8	0.424
Spacing × Likelihood Almost no	–2.2	10.2	–0.2	0.832
Spacing × Likelihood Some	7.0	10.3	0.7	0.493
Semantic Transparency	74.8	8.8	8.5	0.000
Spacing × ST	–6.7	9.1	–0.7	0.458
ST × Likelihood: Almost no	–72.6	9.2	–7.9	0.000
ST × Likelihood: Some	–40.4	9.3	–4.4	0.000
Spacing × ST × Likelihood: Almost no	6.9	12.8	0.54	0.590
Spacing × ST × Likelihood: Some	–1.6	12.9	–0.13	0.898
Constant	5.5	7.1	0.77	0.441
Subjects	2.2	0.07		
Items	1.8	0.10		


#### Discussion

Experiment 2 replicated the effects observed in Experiment 1, including the effects of the semantic distance between the compound and the head. The effects of both LSA and semantic transparency ratings indicate that the modification effect gets larger as the semantic distance between compound and head increases, as one would expect. On the other hand, the inverse modification effect again seems immune to the effects of semantic distance between the compound and the head.

The manipulation of spacing had no impact on the modification or inverse modification effects with the materials in Experiment 2, nor did it affect the way in which the semantic distance interacted with the modification or inverse modification effect. Thus, although spacing can be an important visual cue of whether a compound word actually exists (versus being a novel phrase), when one actually knows of the existence of the compound, spacing is relatively unimportant in inferring properties of the compound.

## Experiment 3

Experiments 1 and 2 showed both modification and inverse modification effects for lexicalized compounds, with a strong asymmetry between the two effects. In addition, we found that the presentation of the compound word (open vs. closed), had no effect on the modification or inverse modification effects with these well-known compound words. This experiment investigates whether these effects depend on the specific knowledge of the known compound word, by replicating Experiment 2, except that we replaced the modifier of the compound words with a non-word (e.g., *blackbird* might become *flegbird*). In particular, the general principle that different names imply real underlying differences (Synonomy Avoidance, Carstairs-McCarthy, 2010; Principle of Contrast, Clark, 1993; and Mutual Exclusivity Principle, Markman, 1989) strongly suggests that manipulations that increase the likelihood of a name appearing to be permanent and unique should affect the extent to which participants infer properties from heads to compounds. The spacing manipulation failed to affect the modification and inverse modification effects in Experiment 2, however. It is possible that this failure is due to the fact that that these compound words are already well known. If so, then unknown words presented as closed compounds should lead to larger effects than unknown words presented as open compounds.

### Methods

#### Participants

Seventy-two participants took part in the experiment. One participant was removed due to a computer problem during data collection. Experiments 1 and 2 had quite large effect sizes, so we set a lower target number of participants at 6 per condition.

#### Materials and Design

The head nouns from the previous experiments were used. Wuggy (Keuleers and Brysbaert, 2010) was used to generate non-words, which then replaced the first constituents of the compounds. The non-words were attached to the head nouns to create fake compounds with realistic compound structure (e.g., *blackbird* could become *flegbird*). The unknown predicates from the previous experiments were used and matched with the same head nouns. The materials were counterbalanced as in the previous experiments. As in Experiment 2, we manipulated whether the concept was modified or unmodified, the likelihood of the property (e.g., Some, Almost All, and Almost No), and whether the compound was presented as open or closed. Modification and Likelihood were within-subject variables and were counterbalanced into 6 lists as in Experiment 1. Spacing was a between-subjects factor to avoid drawing attention to this factor of interest and, thus, there were 12 lists (six with open items and six with closed items). Each participant saw one list. The design was a 2 (Modification) by 3 (Likelihood) by 2 (Spacing) crossed factorial design.

#### Procedure

The procedure was identical to the previous experiments. Because this experiment used non-word modifiers, additional instructions from previous work using non-word modifiers (Spalding and Gagné, 2015) were added. In addition to the task instructions from the previous studies, the participants were told: “When reading, people often come across unfamiliar words. One of our goals is to understand how people interpret phrases and compounds that contain such words. Therefore, in this experiment, some of the items will contain unfamiliar phrases (e.g., flug dogs).”

### Results and Discussion

#### Data Analysis

The descriptive statistics are shown in Table 9. We analyzed the data using LME regression models in which subject and item were entered as crossed random effects, and Modification (modified vs. unmodified), Likelihood (Almost All, Some, Almost No) and Spacing (open vs. closed) were entered as fixed effects, using the mixed and contrast commands in Stata (StataCorp, 2017). The *mixed* function outputs coefficients (i.e., estimates) for simple effects at the first level of the other categorical variables and at the mean of the other continuous variables in the model (see Table 10). For testing our hypotheses, these coefficients are not directly interpretable because they represent the simple effect of a variable at the first level of other variables. The relevant statistical tests for addressing our research questions concern interactions and simple effects, which are reported in the following text. The *contrast* function in Stata was used to conduct these analyses. We report the tests conducted on these fixed effects. Tests of simple effects (the effect of a factor at one level of another factor) were conducted to follow up on statistically significant interactions, because in the case of significant interactions, the main effects are not informative.

**Table 9:** Mean (SE) judged percentage of category members having the test property by level of Likelihood from Experiment 3.

	Percent of category members (*SE*)			
Spacing	Condition	Almost All	Some	Almost No
Closed	Unmodified	91.5 (1.3)	38.8 (2.6)	9.3 (2.5)
	Modified	74.7 (5.0)	33.6 (3.6)	14.1 (3.7)
Open	Unmodified	91.9 (1.7)	39.1 (2.8)	6.9 (1.7)
	Modified	85.7 (3.1)	36.2 (3.3)	9.4 (2.4)


**Table 10:** Experiment 3 Mixed Model Coefficients.

Variable	Coefficient	Standard Error	*z*	*P* > |z|
Spacing	11.1	1.69	6.53	0.000
Likelihood: Almost no	–60.6	0.984	–61.1	0.000
Likelihood: Some	–41.1	0.984	–41.8	0.000
Spacing × Likelihood Almost no	–15.7	1.37	–11.5	0.000
Spacing × Likelihood Some	–8.3	1.37	–6.1	0.000
Modification	16.8	0.984	17.1	0.000
Spacing × Modification	–10.6	1.37	–7.7	0.000
Mod × Likelihood: Almost no	–21.6	1.39	–15.5	0.000
Mod × Likelihood: Some	–11.6	1.39	–8.4	0.000
Spacing × Mod × Likelihood: Almost no	12.9	1.94	6.6	0.000
Spacing × Mod × Likelihood: Some	8.2	1.94	4.2	0.000
Constant	74.6	1.22	61.5	0.000
Subjects	35.1	6.29		
Items	1.29e-09	5.15e-9		


#### Results

The three-way interaction between Spacing, Modification, and Likelihood was significant, χ^2^(2) = 44.99, *p* < 0.0001. To investigate the nature of this interaction, we then carried out separate analyses by level of Likelihood. There was a significant interaction between Modification and Spacing at each level of Likelihood, χ^2^(1) = 65.3, *p* < 0.0001; χ^2^(1) = 4.15, *p* < 0.05; and χ^2^(1) = 4.17, *p* < 0.05 at Almost All, Some, and Almost No, respectively. At each level of Likelihood, the interaction between Modification and Spacing indicated smaller modification (or inverse modification) effects when the item was presented with a space, compared to when it was presented as closed. Unlike Experiment 2, in this case, adding a space clearly attenuated the modification and inverse modification effects, as should be expected if the closed structure is seen by participants as more likely to indicate a permanent, unique name.

Nevertheless, as in the previous experiments, we consistently found modification and inverse modification effects. The tests of the simple effects revealed that the Almost All conditions, both open and closed, led to significant modification effects, *z* = 6.85, *p* < 0.0001 and *z* = 17.95, *p* < 0.0001, respectively. The Some conditions, both open and closed, led to significant modification effects, *z* = 3.59, *p* = 0.001 and *z* = 6.34, *p* < 0.0001, respectively, while the Almost No conditions, both open and closed, led to significant inverse modification effects, *z* = -3.16, *p* = 0.003 and *z* = -5.92, *p* < 0.0001, respectively.

#### Discussion

We once again replicate the robust modification and inverse modification effects, even when the modifiers of the compounds used in the previous experiments are replaced with non-words. Thus, although the previous experiments showed that the semantics of the known compounds contribute to the modification effects, it is clear that the main aspects of the effects are maintained even in cases where no known semantics can be brought to bear on the inference. In addition, unlike Experiment 2, we found that adding a space attenuates both the modification and the inverse modification effect. Presumably, when the participants do not have semantic knowledge to fall back on, they make more use of the visual cue given by the spacing to indicate the permanence of the compound.

## General Discussion

Across all experiments, we found very robust modification (in the Almost All and Some condition) and inverse modification effects (in the Almost No condition) and these effects were observed for both closed and open structures. Thus, although people appear to infer previously unknown properties from heads to compounds, in accordance with a general principle of categorical inference, they make those inferences in line with the general principle that unique names imply underlying semantic differences (as suggested by Synonomy Avoidance, Carstairs-McCarthy, 2010; Principle of Contrast, Clark, 1993; and Mutual Exclusivity Principle, Markman, 1989). In short, when people infer properties from heads to compounds, they do so by coordinating these two general principles regulating the relationship between categories and sub-categories.

In addition, we found that there are two important asymmetries between the modification and inverse modification effects: First, the modification effects are numerically much larger than the inverse modification effects. Second, the size of the modification effect is quite sensitive to the existing semantic distance between a compound word and its head, while the inverse modification effect appears to be entirely insensitive to the existing semantic distance. These results suggest that in making categorical inferences, people are sensitive to the fact that there is an important difference between properties generally true of a category and properties generally untrue of a category, as suggested by previous work on concepts (e.g., Murphy and Medin, 1985). Because our concepts are generally organized around properties that are believed to be true, rather than false, of those concepts, the modification effect (involving properties generally true of the concepts) appears to be much more tightly tied to the existing semantics of the known compounds. In particular, the modification effect seems to be enhanced by the existing differences (when dealing with known compound words). In short, for the modification effect, the more “semantically modified” the compound is, relative to the head, the larger the effect. This result is also consistent with the observation that the more modifiers that are included, the larger the modification effect becomes (Connolly et al., 2007); for example, the modification effect was larger for *Baby Peruvian ducks have webbed feet* than for *Baby ducks have webbed feet*.

The inverse modification effect, on the other hand, seems to be a kind of base line effect that reflects just the general principle that a unique name implies some underlying difference, and seems to be entirely insensitive to the known semantic difference between a compound and its head. Yet, it is sensitive to spacing (in Experiment 3, where the materials are not known compounds), suggesting that the inverse modification effect is sensitive to the likelihood that the “compound” is a permanent, existing word, just not to the semantics of that word.

We have suggested that this pattern of results is consistent with the idea that the results are driven by an underlying conceptual difference between properties considered true of a concept and those considered false. In short, the semantic change that accompanies modification of a concept should be more likely to affect properties true of the head than properties false of the head, on average. We are not, of course, assuming that there are no properties that are true of the compound but false of the head (a well-known example is that pet fish often live in glass bowls, but fish do not usually live in glass bowls). Rather, our point is that there are very many things that are not thought of as true of the head concept (and, indeed, are unrelated to the head) and most of them will remain unrelated to the compound (so, neither fish nor pet fish enjoy salsa dancing, dissolve plastic, or trap dust mites). Thus, any individual, unknown feature that is presented as false of the head, has a relatively strong likelihood of being false of the compound.

Furthermore, features thought of as true of the head concept are often related to each other (see, e.g., Murphy and Medin, 1985), such that a modification of that head that affects one of the features is likely to affect others. For example, if we think of, say, wings, feathers, and flying as being commonly true of birds, we find that a modification that affects one, often affects the others (e.g., birds that cannot fly generally have unusually small wings relative to body size, and their feathers are often quite different from what we think of as “normal” bird feathers—penguin feathers or ostrich feathers, for example). On the other hand, take three things (somewhat randomly) not thought of as true of birds, say explosive, earned a PhD, and made of glass. Now, any of these things might be true of a particular bird in some particular circumstance (a bird used to deliver a bomb strapped to it, a bird given an honorary degree after years of use in a laboratory, or a glass statue of a bird). However, having a compound that affects one of those features (say, an exploding bird), is unlikely to have much consequence for the others; the exploding bird is not particularly likely either to have earned a PhD or to be made of glass.

The point is that properties that are clearly not true of a concept are likely to be very far from the region of semantic space which the head and the compound inhabit, and hence to be unaffected by the relatively small movement in semantic space normally associated with the difference between the head and the compound. In addition, properties that share only the fact that they are false of some concept are likely to be drawn from much more distinct semantic spaces than properties that are all true of that same concept, such that changes to one false property are unlikely to have consequences for other false properties, compared to true properties. Hence, the semantic contrast created by a known compound might be less likely to strongly influence things thought of as false of the head, compared to things thought of as true of the head.

Finally, we found that the presentation format (open or closed) had no effect on the size of the modification and inverse modification effects when the materials were known compound words, but when the modifier of those known compounds was replaced with a non-word, the inclusion of a space attenuated both the modification and inverse modification effects. In general, it seems that when a compound is known to exist, the presentation format does not affect property inference. This is quite reasonable, if the presentation format functions primarily to indicate the higher likelihood of existence as a separate, unique name (when there is no space), because when the compounds are known to exist, the lack of a space does not add to the participants’ certainty that this is a unique name. However, when the modifier of the compound is replaced by a non-word, having the closed structure makes it more likely (in the participants’ view) that the letter string is intended to reflect a permanent, unique name, and hence the modification and inverse modification effects are larger.

An alternative explanation that might occur to the reader is that modification just increases uncertainty, and this explains the modification effects, such that, in essence, participants are simply less likely to use the extremes of the scale. In this view, it is the uncertainty that causes the modification effects (see, e.g., suggestions by Jönsson and Hampton, 2008, 2012; Hampton et al., 2011) rather than the coordination of general principles of categorical inference and of unique names implying underlying semantic differences (Synonomy Avoidance, Carstairs-McCarthy, 2010; Principle of Contrast, Clark, 1993; and Mutual Exclusivity Principle, Markman, 1989). However, uncertainty, though superficially appealing as an explanation, does not explain the details of the effects. First, several aspects of uncertainty were investigated with respect to modification effects in novel compounds, and were found not to account for the effects (Gagné and Spalding, 2014b). Second, presumably, participants would be far more uncertain about properties with respect to novel compounds than known compounds, so an uncertainty explanation predicts that the effects would be larger for novel compounds than for known compounds, but in fact the effects with known compounds are much larger than with novel compounds (e.g., the size of the effects in the current experiments compared to those in Spalding and Gagné, 2015). With respect just to the experiments in the current paper, uncertainty would be far higher with non-word modifiers, so the effects should be much larger with non-word modifiers if those effects are driven by uncertainty, but again this is not the case. Third, uncertainty would be higher for false features (as the relations of such properties even to the head are very unclear, i.e., uncertain), so the effects for the false properties should be larger than for the true properties, but again this is the reverse of the case, both with known compounds in the current experiments and with novel compounds in Spalding and Gagné (2015). Finally, avoiding extreme values on the scale would not explain the modification effect in the “some” condition of the current experiments. Thus, uncertainty seems not to be a good explanation for the overall pattern of the results.

The current results add to the literature on modification effects by showing that the process of property inference is highly consistent across both novel modifications (i.e., conceptual combination) and known compound words, though there are some differences relating to the extent to which the modification is considered to be permanent and unique, and if it is permanent and unique, the extent to which the known compound differs, semantically, from the head. Thus, people know that the purpose of modification is to signal some underlying differences from the unmodified item. The more stable and permanent that modification is believed to be, the more strongly those underlying differences are signaled. Importantly, the modification and inverse modification effects, whether with novel or known compounds, appear to result from inference processes, rather than from a direct, conceptually-driven property inheritance process in which properties of a head are automatically linked to any new modification of that head (see, e.g., Gagné and Spalding, 2011, 2014b, 2015; Spalding and Gagné, 2015, for discussion of this theoretical distinction).

The current results also add to our understanding of the general principle that a unique name implies real underlying differences. An interesting question about the principles of Synonomy Avoidance (Carstairs-McCarthy, 2010), Contrast (Clark, 1993), and Mutual Exclusivity (Markman, 1989) is, what happens once an underlying difference has been identified? Do the principles require simply a minimal difference for each unique name? That is, does knowing of the existence of an underlying difference fully justify the existence of a unique name? If so, then one would expect that items that are already known to differ substantially should be less likely to result in modification (or inverse modification) effects. If there is already a known difference, one need not infer other differences in order to fulfill the needs of Synonomy Avoidance, or Contrast, or Mutual Exclusivity—the compound is already known to differ from the head, so there is no need to create further differences via property inference. Our results strongly suggest that this is not the case (see also Spalding and Gagné, 2015, Experiment 3). Instead, the more the compound is already known to differ from the head, the more it is expected to differ with respect to new, unknown properties, such as those being tested in our experiments. Thus, although the general principle that unique names imply real underlying differences appears to strongly affect property inferences, it is not the case that minimal Synonomy Avoidance, or Contrast, or Mutual Exclusivity is what people are expecting as the result of a unique name. On the other hand, it is also not the case that the unique name implies a need to maximally contrast, such that properties are not inferred at all from the unmodified to the modified. Instead, people appear, generally, to infer properties from the unmodified to the modified noun in an inverse relationship with the degree of contrast that they expect, based on what they know of the modified item, on other generally useful cues such as spacing (when appropriate) or number of modifiers, and on the nature of the property to be inferred (generally true or generally false of the unmodified item).

### Conclusion

This series of experiments indicates that people infer new properties from unmodified nouns to compounds with that noun as the head in accordance with the principle of categorical inference, but also in accordance with the general principle that a unique name implies underlying semantic differences. They appear to make these inferences not in an automatic or mechanical way, but by using the information that they have available to them about the meaning of the compound, the nature of the property (true or false of the head), as well as other cues that they believe are likely to be related to the extent to which the unique name is well established and permanent. Finally, these experiments indicate that property inference follows the same principles, regardless of whether the compound is novel or well known, though the extent to which the compound is believed to be established does affect the degree to which the property is likely to be inferred.

## Ethics Statement

This study was carried out in accordance with the recommendations of the Research Ethics Office of the University of Alberta with written informed consent from all subjects. All subjects gave written informed consent in accordance with the Declaration of Helsinki. The protocol was approved by the Research Ethics Office of the University of Alberta.

## Author Contributions

TS and CG contributed to the theoretical framing, writing, data analysis, and revision. GL contributed to theoretical framing and revision. KN and JC contributed to the data collection and revision. All authors provided critical feedback and helped to shape the research and the manuscript.

## Conflict of Interest Statement

The authors declare that the research was conducted in the absence of any commercial or financial relationships that could be construed as a potential conflict of interest.

## Appendix

**Table A1:** Experimental materials.

Compounds	Predicates
Afterlives	Are dinural
Armpits	Have star shaped carpels
Arrowheads	Have large epiphyseal plates
Barbershops	Use POS software
Bathtubs	Are alloyed with cholorargyrite minerals
Billfolds	Contain casein proteins
Blackbirds	Require graminoids in their diet
Bloodstreams	Have geometric gradients
Blueberries	Contain high amounts of citrulline
Boyfriends	Exhibit dialectic social bonds
Brainwaves	Transmit delta oscillating networks
Bridegrooms	Are studied by malacologists
Broadcasts	Have screens made of polyamid fibers
Busybodies	Have a mobile quadrate bone
Candlesticks	Are used in making Nocello wine
Cheekbones	Secrete the chemical mercaptan
Clamshells	Contain mycoban
Classmates	Are agentic
Cloverleaves	Contain cis-3-hexenal
Coalmines	Are produced with neotame
Corkscrews	Are made from nickel antigorite
Courtyards	Consist of low, tight swards
Crosswords	Are made with combinations of graphemes
Deathtraps	Have a Weberian apparatus
Drawbridges	Have vectualic valves
Dustpans	Contain traces of mixita silica
Fairytales	Come from the Aleutian Islands
Fenceposts	Are built with pneumatic pounders
Fingertips	Are colored by diazo dyes
Floorshows	Became more common when the MFA was bolished
Flowcharts	Represent aeronautical data
Flowerpots	Are cooled in annealing ovens
Footprints	Are lithograph images
Gardenpaths	Have chester webbing
Gemstones	Contain alliin lyase
Goldfinches	Eat Danio rerio
Greyhounds	Have plantigrade paws
Gunpowder	Solidifies after the process of metasomatism
Hailstorms	Contain aromatic hydrocarbons
Hairnets	Are made from jute fibers
Heartburns	Are caused by high concentrations of oleic acid
Hovercrafts	Are facultatively ammonotelic
Inkstands	Have wheels made of cis-1,4-polyisoprene
Jellybeans	Are flavored with strobili
Keyholes	Are caused by structuram dynamics
Lampshades	Are formed from dies with infuse images
Loveboats	Run by compressing the chemical R134a
Madhouses	Follow plazzo hierarchies
Matchboxes	Are lacquered using phenolic compounds
Mincemeat	Has volumetric muscles
Molehills	Have pterylae growths
Moonbeams	Are tetrachromatic
Mothballs	Were first used in episkyros
Necklaces	Have pecten oculi
Notepaper	Is created using cellulose pulp
Paintbrushes	Are susceptible to thrips
Patchwork	Is artisanal
Pawnbrokers	Are descended from mouflons
Photocopiers	Have magnetic solenoids
Placemats	Are made from vulcanized rubber
Plywoods	Contain vitamin HB5
Poppyseeds	Produce purified pectinase
Quicksand	Is made of SiO2
Racehorses	Use vibrissae hairs for tactile sensation
Ribcages	Are secured with helical pieces
Rockpools	Are found in seiches
Rosebuds	Contain a cyanogenic seed
Scarecrows	Are of the genus Loriculus
Scrapbooks	Use the rules of boustrophedon
Seafood	Has cemetrum cells
Sketchpads	Contain protein polymers
Snakeskin	Is made using gliadin proteins
Soupspoons	Were first used in Sardinia
Spacesuits	Come from Witwatersand
Spyglasses	Are used as lorgnettes
Starfish	Swim in a state of tonic immobility
Stomachaches	Are caused by spermaceti
Sugarcanes	Are used by rabologists
Surfboards	Have wooden nocks
Thumbnails	Are sharpened using unite stones
Thunderbolts	Are applied with hexagonal torque
Timetables	Are made of creosote preserved wood
Tinfoil	Is developed using the calotype process
Toothpicks	Have CYL2 materials
Topsoil	Is a part of the pedosphere
Wastebaskets	Are made from baleen
Watermelons	Contain organic apiol
Wheelchairs	Are of ecclesiastical origin
Windpipes	Follow the SMLS structure
Wirecutters	Burn Bitumen based fuels
Witchdoctors	Are artiodactyles
Wristwatches	Have octave key saddles
Airplanes	Use KC-135 quantas
Campfires	Release the chemical HCL
Cavemen	Are of the heterogametic sex
Earthworms	Function with a hydrostatic skeleton
